# A Multiplex Microsphere Immunoassay for Zika Virus Diagnosis

**DOI:** 10.1016/j.ebiom.2017.01.008

**Published:** 2017-01-10

**Authors:** Susan J. Wong, Andrea Furuya, Jing Zou, Xuping Xie, Alan P. Dupuis, Laura D. Kramer, Pei-Yong Shi

**Affiliations:** a Wadsworth Center, New York State Department of Health, Albany, New York, USA; b Department of Biochemistry & Molecular Biology, University of Texas Medical Branch, Galveston, TX, USA; c Department of Pharmacology & Toxicology, University of Texas Medical Branch, Galveston, TX, USA; d Sealy Center for Structural Biology & Molecular Biophysics, University of Texas Medical Branch, Galveston, TX, USA; e Institute for Translational Science, University of Texas Medical Branch, Galveston, TX, USA

**Keywords:** Zika virus, Diagnosis, Serologic assay, Multiplex microsphere immunoassay

## Abstract

Rapid and accurate diagnosis of infectious agents is essential for patient care, disease control, and countermeasure development. The present serologic diagnosis of Zika virus (ZIKV) infection relies mainly on IgM-capture ELISA which is confounded with the flaw of cross-reactivity among different *flaviviruses*. In this communication, we report a multiplex microsphere immunoassay (MIA) that captures the diagnostic power of viral envelope protein (that elicits robust, yet cross-reactive antibodies to other *flaviviruses*) and the differential power of viral nonstructural proteins NS1 and NS5 (that induce more virus-type specific antibodies). Using 153 patient specimens with known ZIKV and/or dengue virus (DENV; a closely related *flavivirus*) infections, we showed that (i) ZIKV envelope-based MIA is equivalent or more sensitive than IgM-capture ELISA in diagnosing ZIKV infection, (ii) antibody responses to NS1 and NS5 proteins are more ZIKV-specific than antibody response to envelope protein, (iii) inclusion of NS1 and NS5 in the MIA improves the diagnostic accuracy when compared with the MIA that uses envelope protein alone. The multiplex MIA achieves a rapid diagnosis (turnaround time < 4 h) and requires small specimen volume (10 μl) in a single reaction. This serologic assay could be developed for use in clinical diagnosis of ZIKV infection and for monitoring immune responses in vaccine trials.

## Highlights

• A multiplex microsphere immunoassay has been developed for Zika virus testing.
• 153 patient specimens used in this assay were PRNT confirmed for the presence or absence of Zika and/or dengue antibodies.
• Antibody responses to viral non-structural proteins were more virus-specific than to viral structural proteins.
• Zika virus diagnosis can be improved by testing for antibodies against non-structural proteins of the virus.

The current epidemic of ZIKV has underlined the importance to develop a rapid and accurate diagnosis. The current serologic diagnosis of ZIKV infection relies mainly upon IgM-capture ELISA which is confounded with the flaw of cross-reactivity among different *flaviviruses*. In this study, we developed a multiplex MIA that captures the diagnostic power of viral envelope protein (that elicits robust, yet cross-reactive antibodies to other *flaviviruses*) and the differential power of viral nonstructural proteins NS1 and NS5 (that induce more virus-type specific antibodies). This serologic assay could be further developed for clinical use and vaccine trials.

## 1 Introduction

Zika virus (ZIKV) belongs to the genus *Flavivirus* within the family *Flaviviridae*. Many *flaviviruses* are significant human pathogens, including ZIKV, yellow fever (YFV), dengue virus (DENV serotypes 1 to 4), Japanese encephalitis virus (JEV), West Nile virus (WNV), and tick-borne encephalitis virus (TBEV). ZIKV is predominantly transmitted by the *Aedes* spp. mosquitoes, which also transmit DENV and YFV, as well as chikungunya virus (an emerging alphavirus). Besides mosquitoes, ZIKV can also be transmitted through maternofetal route, sexual intercourse, blood transfusion, and organ transplantation (Musso and Gubler, 2016). Approximately 80% of the ZIKV infections are asymptomatic. Disease symptoms associated with ZIKV infection include headaches, fever, lethargy, rash, conjunctivitis, myalgia, and arthralgia. Severe diseases of ZIKV infection include neurotropic Guillain-Barre syndrome and congenital microcephaly (Weaver et al. 2016). The *flavivirus* genome is a single-strand, positive-sense RNA of approximately 11,000 nucleotides. It contains a 5′ untranslated region (UTR), an open-reading frame (ORF), and a 3′ UTR. The single ORF encodes a long polyprotein which is processed into ten viral proteins, including three structural proteins [capsid (C), precursor membrane (prM), and envelope (E)] and seven non-structural proteins (NS1, NS2A, NS2B, NS3, NS4A, NS4B, and NS5) (Lindenbach et al., 2013).

Diagnosis of ZIKV infection is performed through detection of viral components (e.g., viral RNA, viral proteins, or virus isolation) and detection of host immune response (e.g., antibodies against viral proteins). For viral component-based diagnosis, RT-PCR, immunoassay, and virus isolation detect ZIKV RNA, viral proteins, and live virus, respectively (Lanciotti et al., 2008). Among them, RT-PCR is the most popular assay because of its sensitivity and specificity, whereas immunofluorescence and ELISA are also commonly used. Indeed, a number of E- and NS1-based assays have been developed for ZIKV diagnosis, including the E-based IgM-captured ELISA from InBios [with Emergency Use Authorization (EUA) approval from FDA], NS1-based indirect ELISA from EuroImmun (approved for clinical use in Europe), and NS1-based IgM-capture ELISA from NovaTec (currently for investigational research use). The viremic phase of ZIKV infection usually lasts for about one week, yet occasionally persists beyond two weeks (Calvet et al., 2016). Due to the short duration of the viremic phase, the diagnostic window for detection of viral components is narrow. Therefore, host immune response-based assays play an important role, among which IgM-capture ELISA (with EUA approval from FDA) and plaque reduction neutralization test (PRNT) are the two most commonly used serologic assays in ZIKV diagnosis. Unfortunately, the interpretation of the current IgM-capture ELISA assays for ZIKV and other *flaviviruses* are challenging due to the cross-reactive nature of antibodies among *flaviviruses*, leading to equivocal diagnostic results. This challenge is confounding Zika diagnosis because (i) many *flaviviruses* (e.g., ZIKV and DENV) produce similar disease symptoms and (ii) antibodies from ZIKV patients cross-react with other *flaviviruses*. Consequently, ZIKV IgM-capture ELISA results typically require neutralization tests for confirmation. Although PRNT remains the gold standard for arbovirus serology, performing PRNT is time consuming, labor intensive with low throughput, and cost-ineffective. Moreover, the PRNT assay still relies upon both virus-specific and cross-reactive epitopes of E protein such that the results could often be inconclusive with respect to *flavivirus* infections (Shan et al., 2016a). Consequently, there is an urgent need to improve the accuracy of the current serologic diagnosis for *flaviviruses*.

Traditionally, serologic assays were designed to detect antibodies against *flavivirus* structural proteins, especially viral E protein in the context of virions. A number of previous studies suggest that antibodies against *flavivirus* nonstructural proteins may be virus-type specific (Garcia et al., 1997, Shu et al., 2002, Wong et al., 2003). These nonstructural proteins could be used to develop more specific serologic assay. More recently, Stettler and colleagues reported virus-type specific NS1 antibodies that were isolated from ZIKV- and DENV-infected patients (Stettler et al., 2016), suggesting that viral NS1 protein should be explored for *flavivirus* serologic diagnosis. The goal of this study was to develop a multiplex microsphere immunoassay (MIA) that detects antibodies against ZIKV structural protein E as well as antibodies to nonstructural proteins NS1 and NS5 to increase the accuracy and speed of diagnosis.

## 2 Materials and Methods

### 2.1 Reagents

Wash buffer and phosphate buffered saline pH 7.4, 0.05% sodium azide (PBS-TN) were purchased from Sigma (Sigma Aldrich, St. Louis, MO). Chemicals, 1-ethyl-3-(3-dimethylaminopropyl) carbodiimide hydrochloride (EDC) and *N*-hydroxysulfosuccinimide (sulfo-NHS), were supplied by Pierce Chemicals (Pierce, Rockford, IL). Microspheres, calibration microspheres, and sheath fluid were obtained from Luminex Corporation (Luminex Corp., Austin, TX).

### 2.2 Serum Samples

Studies were performed on sera from de-identified clinical specimens submitted to New York State Department of Health for ZIKV IgM-capture ELISA and Arbovirus MIA testing [a WNV E protein-based microsphere immunoassay as reported previously (Wong et al., 2003)]. Sera were almost all from returning residents to New York State from travel to the Caribbean, Central America, or South America from the end of 2015 to October of 2016. The demographic profile of this population is approximately 19% Hispanic and 6% Non-Hispanic Asian and Pacific Islander. Many of these individuals may have previous flavivirus immunity, primarily to DENV and other *flaviviruses* as well as YF vaccines. Most sera were collected within two months of travel with possible exposure to ZIKV, but in some instances, patients requested diagnostic tests at later time points. Many individuals were asymptomatic, so the onset dates were not known. The information about patient history with respect to vaccination and previous *flavivirus* infections is not available. The sera from 20 presumed healthy individuals were obtained from the American Red Cross in upstate New York.

### 2.3 Positive and Negative Serum Controls

ZIKV positive control sera were defined as positive titer from a Plaque Reduction Neutralization Test of 90% inhibition (PRNT_90_) against ZIKV, but negative PRNT_90_ titer against DENV. Similarly, DENV positive control sera were defined as positive PRNT_90_ titer against DENV, but negative PRNT_90_ titer against ZIKV. Negative control sera were defined as no ZIKV PRNT_90_ titer as well as negative Arbovirus MIA result using WNV E protein as the diagnostic antigen (Wong et al., 2003). PBN (consisting of PBS, 1% BSA, 0.05% Sodium Azide, pH 7.4) was used as a blank control.

### 2.4 Plaque Reduction Neutralization Test (PRNT)

The PRNT was used as a confirmatory assay to differentiate among recognized *flaviviruses*. ZIKV Puerto Rico strain PRVABC59 and DENV-2 New Guinea strain were used in the PRNT. Briefly, serial dilutions of test samples were mixed with an equal amount of virus suspension containing 200 pfu/0.1 ml and incubated at 37 °C for 1 h. Each virus-diluted serum sample (0.1 ml) was then inoculated onto one well of a 6-well tissue culture plate containing confluent a monolayer of Vero cells. The plate was incubated for 1 h at 37 °C, after which an agar overlay was added and incubation continued. When virus plaques became visible, a second overlay containing neutral red was added and plaques were counted. The antibody titer reported is the dilution of serum that inhibited 90% of the test virus inoculum.

### 2.5 Expression and Purification of Recombinant ZIKA NS5 Protein

The cDNA fragment encoding the full-length NS5 of ZIKV was amplified from an infectious clone pFLZIKV (Shan et al., 2016b), fused with a C-terminal (His)_6_-tag, and cloned into vector pNIC28-Bsa4 (GenBank accession EF198106), resulting in plasmid construct pNIC28-ZIKA-NS5. ZIKA NS5 protein was expressed in *E. coli* Rosetta 2 pLysS *E. coli* (Stratagene) and purified using a method as previously described (Zhao et al., 2015) with some modifications. Briefly, transformed *E. coli* cells were induced by 0.3 mM isopropyl β-d-1-thiogalactopyranoside (IPTG) when the cell density reached OD_600_ of 0.6– 0.8. After incubation at 18 °C for 16 h, the cells were harvested, re-suspended in buffer A (20 mM Tris-HCl, pH 8.5, 550 mM NaCl, 10% glycerol, 5 mM β-mercaptoethanol, 10 mM imidazole, and 0.5 × EDTA-free protease inhibitor cocktail) by sonication. The lysate was clarified by centrifugation at 40,000*g* for 30 min at 4 °C. The resulting supernatant was loaded onto a HisTrap Fast Flow column (GE Healthcare). The protein was eluted using a linear gradient of imidazole concentration from 40 to 500 mM. The fractions containing ZIKA NS5-(His)_6_ protein were pooled, concentrated, and further purified by gel filtration using a HiLoad Superdex 200 16/60 column (GE Healthcare) in buffer B (20 mM Na-Hepes, pH 8.2, 500 mM NaCl, 10% glycerol, and 5 mM DTT). The peak fractions containing ZIKA NS5-(His)_6_ protein were pooled and concentrated to approximately 1– 2 mg/ml before storage at − 80 °C.

### 2.6 Recombinant ZIKV E, NS1, and DENV NS1 Proteins

Recombinant ZIKV E, NS1, and DENV-1 to DENV-4 NS1 proteins were purchased from Meridian (Meridian Life Science, Inc., Memphis, TN). All Meridian recombinant proteins were produced in insect cells and purified by affinity chromatography method. Purified proteins were analyzed by 12.5% sodium dodecyl sulfate (SDS)-polyacrylamide gel electrophoresis (PAGE) and stored in PBS pH 7.4.

### 2.7 Conjugation of Protein Antigens to Microsphere Luminex Beads

Recombinant proteins were covalently coupled to Luminex MicroPlex Microspheres carboxylated polystyrene microparticles following a previously reported protocol (Wong et al., 2003). Briefly, 50 μg of purified protein was used to couple to the surface of 6.25 × 10^6^ microspheres in a two-step carbodiimide process. (i) Activation of microspheres. Microspheres were activated with 10 μl of *N*-hydroxysuccinimide (sulfo-NHS) (50 mg/ml) followed by 10 μl of 1-ethyl-3-(3-dimethylamino-propyl) carbodiimide-HCl (50 mg/ml). Microspheres were then incubated for 20 min at room temperature with gentle vortexing at 10-min intervals. (ii) Coupling of recombinant proteins. Each recombinant protein was added to the activated microspheres with distinct fluorescence. Protein-microsphere mixtures were incubated for 3 h in the dark on a LabTech tube rotator (Barstead/Thermolyne, Dubuque, Iowa). The microspheres were then washed twice by centrifugation and resuspended in 1.0 ml PBS-TN [phosphate buffered saline pH 7.4, 0.05% sodium azide, 1% bovine serum albumin (BSA)]. The protein coupled microsphere were then stored at 4 °C.

### 2.8 Multiplex Reagent Preparation and Microsphere Immunofluorescence Assay (MIA) Procedure

All reagent dilutions and assays were carried out in PBS-TN (phosphate buffered saline pH 7.4, 0.05% sodium azide, 1% BSA) (Sigma Aldrich, St. Louis, MO). The bead mixture consisted of seven beads, each coupled with a different *flavivirus* recombinant protein. Three bead sets contained ZIKV E (Meridian), NS1 (Meridian), and NS5 (described above). Four other bead sets contained NS1 proteins from DENV-1 to -4. The bead sets were stored at 4 °C in the dark and diluted 1:100 directly in PBS-TN before use. Biotin conjugated goat anti-human IgG/A/M affinity purified secondary antibody (Life Technologies, Grand Island, NY) was diluted 1:8000 in PBS-TN directly before use. Streptavidin-R-phycoerythrin (1 mg/ml SA-PE, Life Technologies, Grand Island, NY) was diluted 1:100 in PBS-TN before use. All serum samples were stored at − 80 °C. Samples were thawed and diluted 1:100 in PBS-TN right before use. Diluted samples were used within 1 h post dilution.

A 96-well MultiScreenHTS BV 1.2 μm Filter Plate (Millipore Billerica, MA) was wetted with 100 μl PBS-TN and washed once with washing buffer (PBS, 0.05% Tween 20, pH 7.4; Sigma Aldrich, St. Louis, MO). Samples (50 μl) were dispensed in each well to which 50 μl bead mixture was added. The plate was incubated in the dark on a shaker for 30 min and washed three times with 190 μl washing buffer. After addition of 50 μl conjugate antibody, samples were incubated in the dark on a shaker for 30 min and washed three times with 190 μl wash buffer. After adding detection reagent SA-PE (50 μl), samples were incubated in the dark on a shaker for 30 min, washed twice with 190 μl wash buffer, and transferred to a flat bottom 96-well plate (Corning Incorporated, Kennebunk, ME). Analysis was performed using a Luminex 100 Analyzer configured to count 100 beads per bead class and a 100 μl sample size.

## 3 Results

### 3.1 Rationale for the Assay Platform and Antigen Selection

We chose MIA platform for assay development because of (i) its capability to simultaneously detect antibodies against a number of viral proteins, (ii) low specimen volume requirement (10 μl serum), and (iii) rapid assay turnaround time in < 4 h. This is in contrast with the well-established IgM-capture ELISA that takes > 2 days to complete. With regard to antigens, we selected three recombinant ZIKV proteins for the multiplex assay: E, NS1, and NS5. ZIKV E protein was included to detect *flaviviral* infection; however, due to the cross-reactive nature of E antibodies among *flaviviruses*, an E-positive signal does not confirm ZIKV infection. ZIKV NS1 and NS5 proteins were included to improve assay specificity through detection of virus-type specific antibodies. In addition, recombinant DENV NS1 proteins from each of the four serotypes were included to differentiate between ZIKV and DENV as well as to confirm a potential DENV infection. All seven antigens, with the exception of ZIKV NS5, were commercially purchased. ZIKV NS5 was cloned, expressed, and purified to > 95% homogeneity (Fig.S1).

### 3.2 Establishment of Multiplex MIA

All the above mentioned recombinant antigens (ZIKV E, NS1, NS5, and DENV-1 to -4 NS1) were individually conjugated to microsphere beads, each with a distinct fluorescent signature. A mixture of seven antigen-conjugated beads were reacted with patient serum and quantified by anti-human immunoglobulins (reactive with IgG, IgM, and IgA) with a red fluorescent phycoerythrin. To establish the cutoff level for each antigen, we assayed 20 presumed human sera from healthy individuals in the multiplex MIA. The results revealed cutoff values (defined as mean plus three times standard deviations) to be 1363, 284, 1905, 746, 549, 339, and 655 for ZIKV E, NS1, NS5, and DENV-1, -2, -3, and -4 NS1, respectively. These cutoff values were used to determine positive (> cutoff) and negative (< cutoff) when diagnosing patient specimens. The difference in cut-off values for different proteins might be determined by the intrinsic properties of the proteins.

### 3.3 Stratification of Patient Sera

A well-defined set of patient specimens is essential to develop and verify the multiplex assay. Since PRNT remains the gold standard for *flavivirus* serologic diagnosis, we selected a total of 153 patient sera with known ZIKV and DENV PRNT results for assay development. Positive reactors were defined as a PRNT_90_ antibody titer > 10, while negative reactors had a PRNT_90_ antibody titer < 10. Based on the PRNT_90_ results, we categorized the patient sera into four distinct groups. Group I specimens (7 patients; Table S1) were negative to both ZIKV and DENV. Group II specimens (9 patients; Table S2) were ZIKV-negative and DENV-positive. Group III specimens (42 patients; Table S3) were ZIKV-positive and DENV-negative. Group IV specimens (95 patients; Table S4) were antibody-positive against both ZIKV and DENV. It should be noted that, due to cross-neutralization of antibodies among *flaviviruses*, group IV specimens could derive from either of the following groups of patients: (i) infected with both ZIKV and DENV, (ii) infected with ZIKV only but had antibodies cross-reactive to DENV, or (iii) infected with DENV only but with antibodies cross-reactive to ZIKV.

### 3.4 Multiplex MIA and IgM-Capture ELISA Diagnosis

Patient samples were subjected to multiplex MIA and the well-established IgM-capture ELISA (Martin et al., 2000). Tables S1 through S4 represent the raw data for patient groups I to IV, respectively. Each specimen is presented with results from PRNT, IgM-capture ELISA, and multiplex MIA for individual antigens. It should be pointed out that, for IgM-capture ELISA, P/N < 2 is defined as negative, P/N 2–3 as equivocal, and P/N > 3 as positive; P/N is calculated as the mean optical density (OD) of the test specimen reacted on viral antigen (sucrose-acetone extracted suckling mouse brain viral antigens, provided by CDC) divided by the mean OD of the test specimen reacted on normal antigen (sucrose-acetone extracted suckling mouse brain antigen from mock-infected animals). Table 1 summarizes the overall diagnostic results as follows: for group I specimens (neither ZIKV nor DENV infection), both ZIKV IgM-capture ELISA and E MIA showed 71% negative; the MIA results from ZIKV NS1, ZIKV NS5, and combined DENV-1 to -4 NS1 showed 86% negative (defined as none of the four serotypes of DNEV NS1 was positive). For group II specimens (DENV infection only), ZIKV IgM-capture ELISA showed 67% negative (i.e., 33% cross-reactivity with DENV); ZIKV E and combined DENV-1 to -4 NS1 MIA showed 100% and 89% positive (defined as at least one of the four serotypes of DNEV NS1 was positive), respectively; in contrast, ZIKV NS1 and NS5 MIA showed 78% and 100% negative, respectively. For group III specimens (ZIKV infection only), ZIKV IgM-capture ELISA showed 88% positive; ZIKV E, NS1, and NS5 MIA showed 83%, 100%, and 74% positive, respectively; whereas combined DENV-1 to -4 NS1 MIA showed 64% negative (i.e., 36% cross-reactivity with ZIKV). For group IV specimens (at least one infection from ZIKV and/or DENV), ZIKV IgM-capture ELISA, E, NS1, NS5, and combined DENV NS1 showed 63%, 99%, 100%, 73%, and 96% positive, respectively. These findings enable us to further analyze the results in the context of the following parameters.

**Table 1:** Summary of PRNT, IgM-capture ELISA, and multiplex MIA diagnosis^a^.

Specimen group	Number of specimen	PRNT titer (dilution fold)		ZIKV IgM-capture ELISA (P/N)^b^		ZIKV E^b^	ZIKV NS1^b^	ZIKV NS5^b^	Combined DENV-1 to -4 NS1^b^
		ZIKV	DENV	Equivocal + positive^c^	Positive only^c^				
I	7	< 10	< 10	Negative 5 (5/7 = 71%)	Negative 5 (5/7 = 71%)	Negative 5 (5/7 = 71%)	Negative 6 (6/7 = 86%)	Negative 6 (6/7 = 86%)	Negative 6 (6/7 = 86%)
II	9	< 10	> 10	Negative 6 (6/9 = 67%)	Negative 6 (6/9 = 67%)	Positive 9 (9/9 = 100%)	Negative 7 (7/9 = 78%)	Negative 9 (9/9 = 100%)	Positive 8 (8/9 = 89%)
III	42	> 10	< 10	Positive 39 (39/42 = 93%)	Positive 37 (37/42 = 88%)	Positive 35 (35/42 = 83%)	Positive 42 (42/42 = 100%)	Positive 31 (31/42 = 74%)	Negative 27 (27/42 = 64%)
IV	95	> 10	> 10	Positive 72 (72/95 = 76%)	Positive 60 (60/95 = 63%)	Positive 94 (94/95 = 99%)	Positive 95 (95/95 = 100%)	Positive 69 (69/95 = 73%)	Positive 91 (91/95 = 96%)

a Results from Tables S1 to S4 are summarized for comparison of PRNT, IgM-capture ELISA, and multiplex MIA diagnosis.

b For each diagnostic parameter, the total number of samples that were diagnosed as “positive” (greater than cutoff line) or “negative” (less than cutoff line) is indicated, followed by its corresponding percentage of the total number of specimens from that specific specimen group. Percentage (%) = (number of positive or negative specimens/total number of specimen from the specific specimen group) × 100%.

c Equivocal + positive = the total number of specimens with equivocal (with P/N value between 2 and 3) and positive (with P/N value > 3) IgM-capture ELISA results; positive = number of specimen with P/N value > 3 IgM-capture ELISA results

### 3.5 Comparison of ZIKV IgM-capture ELISA and E MIA

Compared with IgM-capture ELISA, ZIKV E MIA alone showed equivalent accuracy when diagnosing group III specimens (ZIKV only), with 88% and 83% of the samples tested positive from IgM-capture ELISA and E MIA, respectively. When diagnosing group IV specimens (ZIKV and/or DENV), the E MIA showed enhanced sensitivity than IgM-capture ELISA, with 99% and 63% of specimens tested positive, respectively. Two factors may account for this improvement. (i) MIA measures IgG and IgA in addition to IgM, whereas IgM-capture ELISA does not capture IgG and IgA. (ii) The amount of IgM declines after the convalescent phase of ZIKV infection (Russell et al., 2016); therefore, specimens (taken long after convalescent phase) may have low levels of IgM and high levels of IgG, which is no longer detected by the IgM-capture ELISA. Taken together, these results indicate that E MIA alone has equivalent or better sensitivity than IgM-capture ELISA.

### 3.6 Relative Specificity of ZIKV E, NS1, and NS5 MIA

Comparison of the results from ZIKV E, NS1, and NS5 MIA demonstrate that antibody response to NS1 and NS5 antigens is more ZIKV-specific than that to the E antigen. Specifically, ZIKV E MIA showed 100% cross-reactivity with specimens with DENV only infection from group II, confirming the cross-reactive nature of *flavivirus* E antibodies. In contrast, ZIKV NS1 MIA showed 14% and 22% false positive results when testing groups I and II specimens, but 100% positive accuracy when analyzing groups III and IV specimens. For ZIKV NS5 MIA, the assay exhibited 14% and 0% false positive results when testing groups I and II specimens, respectively; and 74% and 73% positive accuracy when analyzing groups III and IV specimens, respectively. The results clearly indicate that inclusion of ZIKV NS1 and NS5 in the MIA could improve the diagnostic accuracy when compared with the MIA that uses the E protein alone.

### 3.7 Cross Reactivity Between DENV/ZIKV NS1 Proteins and their Antibodies

Although antibody response to ZIKV NS1 is more virus-type specific than that to E protein (see above), we clearly observed cross reactivity between DENV and ZIKV NS1 proteins and their antibodies. Specifically, DENV NS1 MIA showed 89% and 96% positive accuracy when testing groups II and IV specimens, respectively; and 14% and 36% false positive results when testing groups I and III specimens, respectively. The 36% false positive result demonstrates that DENV NS1 cross-reacts to specimens with ZIKV-only-infection. Reciprocally, ZIKV NS1 MIA exhibited 22% false positive when testing specimens with DENV-only-infection from group II. Altogether, the results showed 22–36% cross reactivity between DENV and ZIKV NS1 proteins. The data are in agreement with a recent report that antibodies to NS1 are largely ZIKV-specific (Stettler et al., 2016).

## 4 Discussion

The present ZIKV serologic diagnosis is mainly based on IgM-capture ELISA (Lanciotti et al., 2008). The goal of this study was to improve the current serologic diagnosis. Two approaches were taken to achieve this goal. The first approach was to combine the diagnostic power of viral envelope protein (that elicits robust, yet cross-reactive antibodies to other *flaviviruses*) with the differential power of viral nonstructural proteins NS1 and NS5 (that induce the production of more virus-type specific antibodies). The second approach was to develop the assay using an MIA format that can shorten the assay’s turnaround time. The multiplex capability of MIA allowed us to carry out the two approaches, leading to a seven-antigen-based, single well serologic assay. Using 153 patient samples with known ZIKV and DENV PRNT results, we verified the potential of multiplex MIA as an improved serologic diagnosis for ZIKV. Our multiplex MIA is distinct from the single antigen-based (either E or NS1) diagnostic assays that have been recently developed, including the E-based IgM-captured ELISA from InBios, NS1-based indirect ELISA from EuroImmun, and NS1-based IgM-capture ELISA from NovaTec.

Our results indicate that the antibody response to *flavivirus* NS1 is more virus-type specific than to the E protein. These results encourage future studies to identify NS1 epitopes (linear and conformational) that could be used for virus-specific serologic diagnosis. If the virus-specific epitopes are linear, synthetic peptides representing the epitopes could be directly employed by a diagnostic assay; if the virus type-specific epitopes are conformational, the epitopes need to be displayed in a correct structural conformation. Employment of these virus-specific epitopes in the absence of cross-reactive epitopes of NS1 will further improve assay specificity. The same approach could also be applied to identify virus-specific epitopes in NS5 and even E protein. In accordance with this notion, recent studies have shown that domain III of ZIKV E protein contains virus-type specific conformational epitopes (Stettler et al., 2016, Zhao et al., 2016). To further improve the differentiation power of the current assay (i.e., virus-type specificity), DENV NS5 could be added to the current multiplex platform. Since the number of patient specimens was limited in the current study, more well characterized samples are needed to further verify the assay as well as to compare the specificity of antibody responses to NS1 and NS5.

Moving forward, the multiplex capacity of MIA allows one to add more antigens to expand the diagnostic coverage of the assay. Since ZIKV, DENV, WNV, and Chikungunya virus may often co-circulate in the same geographic regions, it would be useful to add antigens that could differentiate infections with these viruses. Compared with ELISA, another advantage of the MIA assay format is its high throughput and low cost (with approximately forty tests per microgram of recombinant protein).

In summary, we report the first multiplex MIA for ZIKV serologic diagnosis that combines viral structural and nonstructural proteins. The MIA platform enables a rapid turnaround time in a multiplex format with improved diagnostic accuracy. The prototype MIA warrants further development for clinical diagnosis of ZIKV infection as well as for monitoring immune response in vaccine trial.

## Funding Sources

P.Y.S. was supported by University of Texas Medical Branch (UTMB) startup award, UTMB Innovation and Commercialization award, University of Texas STARs Award, NIH grant R01AI087856, Pan American Health Organization grant SCON2016-01353, and UTMB CTSA UL1TR-001439.

## Conflict of Interest Statement

The authors have no conflict of interest in this study.

## Author Contributions

A.F., J.Z., X.X., and APD performed experiments and data analysis. S.J.W., A.F., J.Z., K.D., and P.Y.S. interpreted the results. S.J.W., A.F., J.Z., and P.Y.S. wrote the manuscript.

## Appendix A Supplementary data

Supplementary material Image 1

## References

[bb0005] Calvet G.A., Santos F.B., Sequeira P.C. (2016). Zika virus infection: epidemiology, clinical manifestations and diagnosis. Curr. Opin. Infect. Dis..

[bb0010] Garcia G., Vaughn D.W., Del Angel R.M. (1997). Recognition of synthetic oligopeptides from nonstructural proteins NS1 and NS3 of dengue-4 virus by sera from dengue virus-infected children. Am.J.Trop. Med. Hyg..

[bb0015] Lanciotti R.S., Kosoy O.L., Laven J.J., Velez J.O., Lambert A.J., Johnson A.J., Stanfield S.M., Duffy M.R. (2008). Genetic and serologic properties of Zika virus associated with an epidemic, Yap State, Micronesia, 2007. Emerg. Infect. Dis..

[bb0020] Lindenbach B.D., Murray C.L., Thiel H.J., Rice C.M., Knipe D.M., Howley P.M. (2013). Flaviviridae. Fields Virology.

[bb0025] Martin D.A., Muth D.A., Brown T., Johnson A.J., Karabatsos N., Roehrig J.T. (2000). Standardization of immunoglobulin M capture enzyme-linked immunosorbent assays for routine diagnosis of arboviral infections. J. Clin. Microbiol..

[bb0030] Musso D., Gubler D.J. (2016). Zika virus. Clin. Microbiol. Rev..

[bb0035] Russell K., Oliver S.E., Lewis L., Barfield W.D., Cragan J., Meaney-Delman D., Staples J.E., Fischer M., Peacock G., Oduyebo T. (2016). Update: interim guidance for the evaluation and management of infants with possible congenital Zika virus infection - United States, August 2016. MMWR Morb. Mortal. Wkly Rep..

[bb0040] Shan C., Xie X., Barrett A.D.T., Garcia-Blanco M.A., Tesh R.B., Vasconcelos P.F.D.C., Vasilakis N., Weaver S.C., Shi P.Y. (2016). Zika virus: diagnosis, therapeutics, and vaccine. ACS Infect. Dis..

[bb0045] Shan C., Xie X., Muruato A.E., Rossi S.L., Roundy C.M., Azar S.R., Yang Y., Tesh R.B., Bourne N., Barrett A.D. (2016). An infectious cDNA clone of Zika virus to study viral virulence, mosquito transmission, and antiviral inhibitors. Cell Host Microbe.

[bb0050] Shu P.Y., Chen L.K., Chang S.F., Yueh Y.Y., Chow L., Chien L.J., Chin C., Yang H.H., Lin T.H., Huang J.H. (2002). Potential application of nonstructural protein NS1 serotype-specific immunoglobulin G enzyme-linked immunosorbent assay in the seroepidemiologic study of dengue virus infection: correlation of results with those of the plaque reduction neutralization test. J. Clin. Microbiol..

[bb0055] Stettler K., Beltramello M., Espinosa D.A., Graham V., Cassotta A., Bianchi S., Vanzetta F., Minola A., Jaconi S., Mele F. (2016). Specificity, cross-reactivity, and function of antibodies elicited by Zika virus infection. Science.

[bb0060] Weaver S.C., Costa F., Garcia-Blanco M.A., Ko A.I., Ribeiro G.S., Saade G., Shi P.Y., Vasilakis N. (2016). Zika virus: history, emergence, biology, and prospects for control. Antivir. Res..

[bb0065] Wong S.J., Boyle R.H., Demarest V.L., Woodmansee A.N., Kramer L.D., Li H., Drebot M., Koski R.A., Fikrig E., Martin D.A. (2003). An immunoassay targeting nonstructural protein 5 to differentiate West Nile virus infection from dengue and St. Louis encephalitis virus infections, and form flavivirus vaccination. J. Clin. Microbiol..

[bb0070] Zhao Y., Soh T.S., Chan K.W., Fung S.S., Swaminathan K., Lim S.P., Shi P.Y., Huber T., Lescar J., Luo D. (2015). Flexibility of NS5 methyltransferase-polymerase linker region is essential for dengue virus replication. J. Virol..

[bb0075] Zhao H., Fernandez E., Dowd K.A., Speer S.D., Platt D.J., Gorman M.J., Govero J., Nelson C.A., Pierson T.C., Diamond M.S. (2016). Structural basis of Zika virus-specific antibody protection. Cell.

