# Supplementary material for: A Multiplex Microsphere Immunoassay for Zika Virus Diagnosis
Source: eBioMedicine. 2017 Jan 10;16:136–40. doi: 10.1016/j.ebiom.2017.01.008 (PMC5474433; doi:10.1016/j.ebiom.2017.01.008)
Supplement: Supplementary file 1 — Supplementary material [file mmc1.pdf]

Supplemental figure

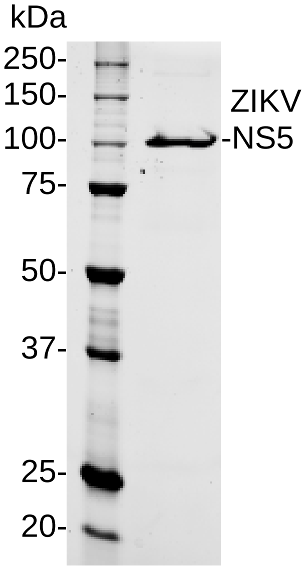

Figure S1. SDS-PAGE analysis of recombinant ZIKV NS5 protein. Full-length NS5 of ZIKV was expressed in an *E.coli* system, and purified through affinity followed by size-exclusion chromatography (see details in Materials and Methods). The recombinant protein was analyzed on a 12% Mini-PROTEAN® TGX Stain-Free™ Protein Gel (Bio-rad).

Table S1. Group specimens with neither ZIKV nor DENV infection defined by PRNT*

<table>
<tr><th>ZIKV PRNT Cutoff 10</th><th>DENV PRNT Cutoff 10</th><th colspan="2">ZIKV IgM-capture ELISA P/N 2 & 3</th><th>ZIKV E Cutoff 1363</th><th>ZIKV NS1 Cutoff 284</th><th>ZIKV NS5 Cutoff 1905</th><th>DENV-1 NS1 Cutoff 746</th><th>DENV-2 NS1 Cutoff 549</th><th>DENV-3 NS1 Cutoff 339</th><th>DENV-4 NS1 Cutoff 655</th></tr>
<tr><td><10</td><td><10</td><td colspan="2">0.2</td><td>1189</td><td>167</td><td>573</td><td>1200</td><td>935</td><td>100</td><td>450</td></tr>
<tr><td><10</td><td><10</td><td colspan="2">0.5</td><td>1415</td><td>155</td><td>308</td><td>220</td><td>259</td><td>61</td><td>96</td></tr>
<tr><td><10</td><td><10</td><td colspan="2">0.9</td><td>195</td><td>54</td><td>1128</td><td>87</td><td>51</td><td>230</td><td>175</td></tr>
<tr><td><10</td><td><10</td><td colspan="2">1.9</td><td>366</td><td>91</td><td>1900</td><td>306</td><td>216</td><td>168</td><td>136</td></tr>
<tr><td><10</td><td><10</td><td colspan="2">1.9</td><td>7742</td><td>4619</td><td>4267</td><td>127</td><td>105</td><td>113</td><td>275</td></tr>
<tr><td><10</td><td><10</td><td colspan="2">3.8</td><td>178</td><td>69</td><td>534</td><td>235</td><td>141</td><td>113</td><td>211</td></tr>
<tr><td><10</td><td><10</td><td colspan="2">11.6</td><td>1001</td><td>53</td><td>211</td><td>418</td><td>509</td><td>75</td><td>125</td></tr>
<tr><td colspan="2">Positive sample number /total sample number</td><td>P/N >2: 2/7</td><td>P/N >3: 2/7</td><td>2/7</td><td>1/7</td><td>1/7</td><td colspan="4">1[#]/7</td></tr>
</table>

*The table is sorted with increasing value of IgM-capture ELISA result. The cutoff values for each assay are indicated.

[#]Specimens with at least one serotype of DENV NS1-positive are considered as “positive.”

Table S2. Group II specimens with DENV only infection defined by PRNT*

<table>
<tr><th>ZIKV PRNT Cutoff 10</th><th>DENV PRNT Cutoff 10</th><th colspan="2">ZIKV IgM-capture ELISA Cutoff 2 & 3</th><th>ZIKV E Cutoff 1363</th><th>ZIKA NS1 Cutoff 284</th><th>ZIKV NS5 Cutoff 1905</th><th>DENV-1 NS1 Cutoff 746</th><th>DENV-2 NS1 Cutoff 549</th><th>DENV-3 NS1 Cutoff 339</th><th>DENV-4 NS1 Cutoff 655</th></tr>
<tr><td><10</td><td>20</td><td colspan="2">0.19</td><td>9131</td><td>234</td><td>170</td><td>5375</td><td>1579</td><td>3091</td><td>1442</td></tr>
<tr><td><10</td><td>40</td><td colspan="2">0.60</td><td>7145</td><td>211</td><td>331</td><td>9101</td><td>2831</td><td>5415</td><td>3740</td></tr>
<tr><td><10</td><td>80</td><td colspan="2">1.5</td><td>11139</td><td>2514</td><td>249</td><td>9145</td><td>6658</td><td>10609</td><td>14941</td></tr>
<tr><td><10</td><td>160</td><td colspan="2">1.5</td><td>9596</td><td>1806</td><td>177</td><td>7486</td><td>5198</td><td>8241</td><td>11867</td></tr>
<tr><td><10</td><td>640</td><td colspan="2">1.5</td><td>5381</td><td>46</td><td>227</td><td>5323</td><td>860</td><td>2235</td><td>1352</td></tr>
<tr><td><10</td><td>80</td><td colspan="2">1.6</td><td>8071</td><td>67</td><td>554</td><td>6862</td><td>510</td><td>1765</td><td>658</td></tr>
<tr><td><10</td><td>40</td><td colspan="2">5.1</td><td>6017</td><td>51</td><td>1473</td><td>98</td><td>50</td><td>99</td><td>165</td></tr>
<tr><td><10</td><td>160</td><td colspan="2">5.8</td><td>7756</td><td>38</td><td>968</td><td>4259</td><td>179</td><td>582</td><td>305</td></tr>
<tr><td><10</td><td>40</td><td colspan="2">77</td><td>8703</td><td>106</td><td>1009</td><td>8805</td><td>1511</td><td>3165</td><td>1558</td></tr>
<tr><td colspan="2">Positive sample number /total sample number</td><td>P/N >2: 3/9</td><td>P/N >3: 3/9</td><td>9/9</td><td>2/9</td><td>0/9</td><td colspan="4">8[#]/9</td></tr>
</table>

*The table is sorted with increasing value of IgM-capture ELISA result. The cutoff values for each assay are indicated.

[#]Specimens with at least one serotype of DENV NS1-positive are considered as "positive."

Table S3. Group III specimens with ZIKV only infection defined by PRNT *

ZIKV PRNT Cutoff 10	DENV PRNT Cutoff 10	ZIKV IgM-capture ELISA Cutoff 2 & 3	ZIKV E Cutoff 1363	ZIKA NS1 Cutoff 284	ZIKV NS5 Cutoff 1905	DENV-1 NS1 Cutoff 746	DENV-2 NS1 Cutoff 549	DENV-3 NS1 Cutoff 339	DENV-4 NS1 Cutoff 655
80	<10	0.6	9960	5207	2624	1346	1172	454	744
320	<10	0.99	10692	6173	1676	409	531	660	631
2560	<10	1.8	16318	9407	10423	548	737	802	492
40	<10	2.4	858	617	1401	349	272	249	374
10	<10	2.75	2185	297	1777	795	729	189	285
80	<10	3.04	800	481	227	163	213	118	252
160	<10	3.80	9088	7010	3298	438	588	782	459
320	<10	3.97	11272	4849	2129	1556	1268	250	838
320	<10	4	11358	4933	2179	1503	1212	212	735
320	<10	4.5	8592	5500	4362	311	455	452	220
160	<10	6.2	3896	3152	2524	574	477	203	371
160	<10	7.88	5782	701	574	340	260	125	217
1280	<10	7.9	3233	5196	5865	231	263	315	246
1280	<10	7.9	4777	6771	8260	364	417	508	381
320	<10	8.1	8842	4811	15599	214	349	561	276
320	<10	8.1	9768	5001	18463	253	405	532	294
320	<10	8.13	12377	6550	21510	391	644	927	422
160	<10	8.29	457	540	930	464	258	204	168
1280	<10	10.9	496	1010	3139	102	84	74	108
160	<10	13.5	12420	7611	1809	141	195	218	401
160	<10	13.5	14292	8757	2491	180	243	265	548
160	<10	13.7	7432	6664	2876	90	159	337	149
40	<10	17	536	1554	2612	153	202	122	153
640	<10	17.70	6381	7713	18520	1095	1082	628	419
640	<10	20.1	2474	2312	2038	197	205	67	82
640	<10	20.18	3703	1566	4269	75	69	314	54

<table>
<tr><td>160</td><td><10</td><td colspan="2">22</td><td>530</td><td>428</td><td>679</td><td>151</td><td>116</td><td>110</td><td>119</td></tr>
<tr><td>160</td><td><10</td><td colspan="2">22</td><td>999</td><td>5442</td><td>3802</td><td>217</td><td>234</td><td>263</td><td>86</td></tr>
<tr><td>320</td><td><10</td><td colspan="2">25</td><td>7905</td><td>6814</td><td>5828</td><td>244</td><td>350</td><td>242</td><td>166</td></tr>
<tr><td>320</td><td><10</td><td colspan="2">25.5</td><td>8518</td><td>6914</td><td>6650</td><td>261</td><td>378</td><td>303</td><td>228</td></tr>
<tr><td>1280</td><td><10</td><td colspan="2">27</td><td>8494</td><td>5334</td><td>3631</td><td>102</td><td>107</td><td>103</td><td>274</td></tr>
<tr><td>1280</td><td><10</td><td colspan="2">27.18</td><td>10443</td><td>6998</td><td>4530</td><td>157</td><td>163</td><td>158</td><td>407</td></tr>
<tr><td>640</td><td><10</td><td colspan="2">28</td><td>3488</td><td>11213</td><td>4269</td><td>290</td><td>452</td><td>579</td><td>62</td></tr>
<tr><td>640</td><td><10</td><td colspan="2">28.8</td><td>8741</td><td>7471</td><td>3820</td><td>278</td><td>492</td><td>662</td><td>147</td></tr>
<tr><td>640</td><td><10</td><td colspan="2">29.9</td><td>6212</td><td>4915</td><td>5969</td><td>193</td><td>221</td><td>288</td><td>115</td></tr>
<tr><td>640</td><td><10</td><td colspan="2">30</td><td>3998</td><td>3187</td><td>3867</td><td>133</td><td>141</td><td>158</td><td>73</td></tr>
<tr><td>1280</td><td><10</td><td colspan="2">32</td><td>11745</td><td>7674</td><td>4459</td><td>174</td><td>49</td><td>242</td><td>250</td></tr>
<tr><td>1280</td><td><10</td><td colspan="2">32.3</td><td>13259</td><td>8154</td><td>4726</td><td>227</td><td>309</td><td>304</td><td>309</td></tr>
<tr><td>640</td><td><10</td><td colspan="2">42.1</td><td>9721</td><td>7456</td><td>658</td><td>143</td><td>276</td><td>230</td><td>139</td></tr>
<tr><td>2560</td><td><10</td><td colspan="2">47.2</td><td>16346</td><td>4928</td><td>3714</td><td>149</td><td>158</td><td>320</td><td>60</td></tr>
<tr><td>320</td><td><10</td><td colspan="2">50.4</td><td>8250</td><td>7160</td><td>638</td><td>134</td><td>251</td><td>215</td><td>122</td></tr>
<tr><td>320</td><td><10</td><td colspan="2">65.6</td><td>3645</td><td>2785</td><td>1229</td><td>52</td><td>74</td><td>77</td><td>79</td></tr>
<tr><td colspan="2">Positive sample number /total sample number</td><td>P/N>2: 39/42</td><td>P/N >3: 37/42</td><td>35/42</td><td>42/42</td><td>31/42</td><td colspan="4">15[#]/42</td></tr>
</table>

*The table is sorted with increasing value of IgM-capture ELISA result. The cutoff values for each assay are indicated.

[#]Specimens with at least one serotype of DENV NS1-positive are considered as “positive.”

Table S4. Group specimens with ZIKV and/or DENV infection(s) defined by PRNT*

ZIKV PRNT Cutoff 10	DENV PRNT Cutoff 10	ZIKV IgM-capture ELISA Cutoff 2 & 3	ZIKV E Cutoff 1363	ZIKV NS1 Cutoff 284	ZIKV NS5 Cutoff 1905	DENV-1 NS1 Cutoff 746	DENV-2 NS1 Cutoff 549	DENV-3 NS1 Cutoff 339	DENV-4 NS1 Cutoff 655
2560	320	0.3	12411	3002	6118	7992	5677	11340	10357
10	40	0.40	7446	285	790	7714	2875	5725	4420
320	2560	0.4	15916	4164	11951	10070	8345	14047	13683
20	1280	0.41	18428	802	461	7969	5627	10631	9581
80	640	0.45	17887	2203	665	5073	3700	5778	9545
160	640	0.45	18357	2550	796	5886	4181	6677	10700
20	640	0.45	17361	874	551	9206	6339	11251	11171
10	80	0.49	16355	1950	570	10920	8275	15354	11740
1280	2560	0.53	18809	6682	650	16472	10777	18272	19435
10	160	0.53	8131	844	325	5142	3838	7463	6020
1280	2560	0.56	20863	6548	602	16500	10369	19309	19308
160	2560	0.62	17472	3453	2503	12655	2806	11109	16787
160	2560	0.69	18220	3461	2384	13241	2871	12265	17857
2560	640	0.8	18354	8434	2396	382	426	556	309
640	1280	1.09	16382	4065	3303	11394	7116	13885	7695
320	2560	1.3	17322	4085	875	15507	11175	17637	16700
2560	320	1.41	19995	6926	8456	12524	11268	17951	16740
640	1280	1.53	21501	7952	3037	16148	12733	19792	22445
320	160	1.63	14009	6104	1374	11801	11004	17719	4475
320	2560	1.7	19788	8615	3494	16160	13589	19342	20432
160	2560	1.8	16928	3607	551	14462	10561	17158	15002
1280	1280	1.81	17896	7868	2698	14294	11672	17853	21180
320	2560	1.9	20374	8309	2755	16466	13975	19997	20689
640	2560	2.1	21021	12154	15783	18993	14796	21073	22419
640	2560	2.16	21035	11444	13536	18240	15675	21260	22412
5160	5120	2.3	18554	6707	553	12751	10464	17362	20153

5120	1280	2.3	19606	11677	11105	14953	11886	18231	21051
5120	1280	2.32	18697	9335	7705	12257	9981	16633	17776
640	1280	2.45	20559	10939	21381	14675	10696	18537	17415
2560	1280	2.5	19945	9641	13280	15589	13022	19484	15561
2560	1280	2.52	18959	8035	11647	14771	12827	19260	13601
320	40	2.57	9760	1309	386	5967	1610	2724	3719
160	40	2.59	10893	859	180	3100	914	1643	6687
160	40	2.72	13602	1082	205	4179	1260	2191	8609
320	40	2.99	10133	1442	417	6469	1696	3018	4257
320	5120	3.13	18591	7977	1050	11995	9617	15868	14587
160	320	3.53	19680	5587	1681	317	292	543	270
2560	2560	3.89	17268	4501	5464	13748	9869	17527	12169
5120	5120	3.99	21644	10859	21825	12761	9859	17755	20283
160	320	4.02	16822	6103	2070	14021	10298	17324	15517
640	1280	4.42	20593	5338	1739	12752	11158	18128	18042
1280	1280	4.43	19973	8594	11143	12763	11051	17739	16997
1280	320	4.79	19355	10636	9796	12751	10778	18003	18073
640	1280	4.88	16101	4858	776	13093	8198	14431	8430
2560	1280	5.01	20780	12080	17177	16920	13760	20359	17012
160	640	5.1	19275	6311	2744	14623	12984	19646	15016
160	640	5.34	16289	5747	2635	15009	11904	18826	16649
10	80	5.47	16794	1307	1328	389	200	346	281
1280	640	6.05	16809	6987	3429	13666	12416	18579	5716
1280	80	6.52	2111	3626	1100	209	289	243	291
320	160	7.04	19585	2905	3390	10849	5579	10368	11556
320	160	7.04	20334	3419	4461	11917	6275	12136	12769
320	80	7.32	19940	2687	2942	10583	5368	9833	10471
1280	10	7.49	13338	9654	6618	462	650	1220	700
1280	10	7.5	12624	9195	6717	341	478	986	514
2560	20480	8.3	20265	9171	2720	16781	12747	18712	20473

640	5120	8.5	20671	9098	7078	17100	14388	18608	22061
640	10240	9.04	21114	11671	11204	19358	15223	21543	22106
2560	320	10.16	18279	9899	7213	4524	3165	6948	1278
160	640	10.90	20183	7660	2078	2723	2628	5909	1384
320	640	10.99	14737	4336	15626	11828	9458	15927	6841
320	2560	11.1	20977	10041	2254	17295	13477	19549	10638
640	10240	11.4	20785	12169	11619	18884	15119	20667	21989
1280	2560	12	19968	8988	4259	17044	13238	19273	22230
1280	2560	12.54	20467	8901	3673	16470	13221	19341	22846
320	640	13	20546	6045	1818	15929	10974	18017	3192
640	1280	14	18531	5202	1896	11359	10329	16770	16440
640	640	14.64	20336	7675	1722	13716	12002	18849	20055
320	2560	15	21156	10463	3187	18463	14572	20246	22876
640	2560	16	20527	9862	5041	16141	12408	19080	21991
640	2560	16.29	19340	9066	5185	15029	11748	17407	20104
2560	20480	17	22317	6665	7447	15855	10725	17403	5509
2560	20480	17.23	17999	5738	7214	13635	8930	14737	5748
160	1280	17.41	19848	8375	6839	15679	13745	18998	22484
160	160	20.73	9391	6074	3340	356	679	566	309
10240	320	22	17752	7270	4264	62	156	388	96
1280	1280	22.2	20185	8584	16360	16030	12594	20100	10783
1280	20480	23	20703	10096	7859	18086	13614	18507	21267
320	20	23.6	9924	8081	9837	113	203	469	231
1280	5120	23.69	18836	8498	4119	15799	12910	18325	20333
320	10	25	1061	3108	2294	88	89	83	894
640	640	25.23	20066	9245	4529	17030	14304	20372	22431
2560	2560	26.13	21184	9524	2734	15615	13845	19053	22401
640	2560	27.9	19121	3787	3230	3522	3308	7426	3591
1280	10240	29.15	19711	10327	2437	16973	14262	18898	20341
1280	2560	29.9	21902	8590	3728	16006	13452	20836	22510

640	1280	30.53	18080	8675	17545	16414	13525	18677	14452
2560	640	32	20207	6094	11566	10231	7930	14480	11206
640	2560	33.4	20649	10126	6628	16806	13194	18759	22293
320	160	35.88	4640	4268	2211	294	263	175	169
320	10	44.8	9837	7200	2590	284	253	308	194
2560	2560	46.1	19943	7114	7170	15766	11664	18454	8917
640	640	47.93	2828	5437	7229	334	1185	1407	299
1280	20	49.11	14063	6262	6839	359	291	193	250
1280	640	57	19062	6018	11756	12258	10914	17240	17024
**Positive sample number /total sample number**		P/N >2: 72/95<br>P/N >3: 60/95	94/95	95/95	69/95	91[#]/95			

*The table is sorted with increasing value of IgM-capture ELISA result. The cutoff values for each assay are indicated.

[#]Specimens with at least one serotype of DENV NS1-positive are considered as “positive.”
